# Perceptions of teachers towards COVID appropriate behaviors for school children in coastal South India

**DOI:** 10.1007/s12144-021-02564-z

**Published:** 2022-01-10

**Authors:** Sowmini Padmanabh Kamath, Prasanna Mithra, Jayateertha Joshi, Padmanabh Kamath, Bhaskaran Unnikrishnan, Jayashree K., Suchetha Rao, Ravikiran S.R., Keshav Pai, Nutan Kamath, Kamalakshi G. Bhat

**Affiliations:** 1 Department of Pediatrics, Kasturba Medical College, Mangalore, Manipal Academy of Higher Education, Manipal, India; 2 Department of Community Medicine, Kasturba Medical College, Mangalore, Manipal Academy of Higher Education, Manipal, India; 3 Department of Pediatric Surgery, Kasturba Medical College, Mangalore, Manipal Academy of Higher Education, Manipal, India; 4 Department of Cardiology, Kasturba Medical College, Mangalore, Manipal Academy of Higher Education, Manipal, India; 5 Department of Psychiatry, Kasturba Medical College, Mangalore, Manipal Academy of Higher Education, Manipal, India

**Keywords:** Child, Counselors, Hygiene, Perception, Physical distancing, Psychological stress, School teachers

## Abstract

COVID appropriate behavioral measures need to be followed once school reopens. School teachers being in the forefront could substantiate the feasibility of suggested safety measures. This study aimed to assess teachers’ perceptions towards COVID appropriate behaviors for children with school reopening and compare their mean scores between public versus private schools and across school boards. We conducted an observational school-based study of teachers over two months. Perceptions were scored using a five-point Likert symmetric agree to disagree scale. Results were expressed as proportions and analyzed using an independent sample t-test. Of the 547 teachers surveyed, most (> 90%) agreed to the suggested social distancing and hygiene measures. There was a significant difference in perception scores between private versus public schools and across boards regarding i) reducing the academic syllabus, ii) adopting a cloud-based system to integrate online-offline learning, and iii) conducting meetings online. In addition, measures such as i) teaching classes on alternate days with a limited number of children, ii) arranging benches/desks to maintain six feet distance between students, iii) dealing with psychological stress by counselors, and iv) arrangement with local hospitals for medical services were significant statistically across school boards. To conclude, most schoolteachers agreed with the need for social distancing and hygiene measures for children. There was a significant difference in perceptions between public versus private schools and across boards regarding academic syllabus, integration of online-offline student learning, number of children per class, the timing of classes, student seating arrangement, and medical/psychological guidance availability.

## Introduction

World Health Organization declared the outbreak of the 2019 novel coronavirus disease (COVID-19) as a pandemic on March 11, 2020. (World Health Organization [WHO], 2020a). To contain the spread of COVID 19 infection, lockdown measures were implemented in many countries, resulting in the temporary closure of educational institutions, which has influenced over 154 crore students, 32 crores in India (PTI, 2020). The COVID-19 pandemic has been responsible for the most considerable disruption of education systems in history in over 190 countries. It has affected about 94% of the world’s student population and affected 99% in low and middle-income countries (United Nations [UN], 2020). The Indian educational institutes continued to be closed because of the second wave of COVID 19 and have currently opened in a phased manner.

Due to uncertainties regarding the duration of school closures, schoolteachers had to switch over and adapt from the traditional face-to-face to online teaching mode to have continued education for children and manage extra responsibilities of their families as well (Dhawan, 2020; United Nations Educational Scientific and Cultural Organization [UNESCO], 2020a). Online education may not be well suited for some children, and teachers need to be more creative to have children under attention. School closures also influenced the children vastly since they experienced a lack of social interaction with their peers and teachers, which is essential for their social development. In addition, adjusting to online teaching methods, inadequate housing, and issues with internet data, especially in developing countries, affected their education. Similarly, insecurities in family finances, food shelter, and increasing unemployment also had affected many families (UNESCO, 2020a).

Worldwide media coverage and information by the Government of India has influenced the adoption of social distancing and hygiene strategical measures among the public over the months (Centers for Disease Control and Prevention [CDC], 2020; Ministry of Health and family welfare, Government of India [MoHFW, (GOI)], 2020; WHO, 2020b). Challenges will arise in specific populations and atmospheres, such as the school atmosphere. With the reopening of schools, teachers being the frontline forces have additional responsibilities to maintain adherence to infection preventive and control measures. At the same time, they need to ensure their students are exercising the same standards effectively to control disease spread.

The preventive measures at the school/workplace must include personal preventive measures (e.g., hand hygiene, social distancing, wearing a facemask, and others). In addition, it should follow school organizational measures (e.g., social distancing at various areas in school, distance learning using technology, good ventilation, etc.) for smooth functioning (Kim & Su, 2020).

During the influenza pandemic, transmission surges with an increase in influenza-like illness (ILI) once school reopened had been seen (Chao et al., 2010; Cowling et al., 2008). Thus, with school reopening, there is a fear of the increased risk of resurgence of COVID 19 infection among the public.

Working in a new normal situation during the COVID 19 requires strategic planning to have appropriate safety measures at all educational institutions to safeguard children, teachers, and support staff. Various organizations like UNICEF, UNESCO, WHO, Ministry of education, India (Inter-Agency Standing Committee [IASC], 2020; UNESCO, 2020b; The Ministry of Home Affairs, 2020), have chalked out various measures for smooth functioning of school along with learning activities. With school reopening, getting into the stream of having face-to-face interactive classes with maintaining COVID 19 appropriate behaviors will be another challenge for the teachers, children, and their families.

The high implementation rate of Taiwanese teachers’ disease prevention measures came from their higher risk perceptions (Tang et al., 2021). Preventive behavior and risk perceptions have been studied in various populations, namely in Turkish health care workers (Arslanca et al., 2021), university students (Vande Velde et al., 2021; Mant et al., 2021), and Australian adults (Seale et al., 2020). The COVID 19 snapshot monitoring (COSMO) study demonstrated differences in the risk perception, knowledge, and protective behavior regarding COVID 19 based on the education levels of German men and women (Rattay et al., 2021).

Understanding teachers’ perceptions, who are at the forefront, would substantiate the feasibility of implementing regulations on social distancing and hygiene measures and thereby help in the policymaking. Hence, we conducted this study to assess teachers’ perceptions towards COVID appropriate behaviors for managing children once school reopens. Further, we compared their perceptions between private and public schools and across school boards.

## Methodology

### Participants and Setting

After approval from Institutional Ethics Committee (IEC), we did an observational school-based cross-sectional study of school teachers in selected schools of Dakshina Kannada district over two months duration. Assuming that 50% of teachers would have positive perceptions towards COVID-appropriate behaviors with 10% relative precision, 95% confidence interval, applying finite population correction, and 10% non-response rate, we calculated the sample size to be 306.

We obtained a list of all the schools within the Dakshina Kannada district from the Deputy Director of Public Instructions (DDPI) office. Using simple random sampling, we divided the sample size equally among the public and private schools in each category. We listed the schools in the ascending order of teachers’ strengths. Using the lottery technique, we selected the schools until we could achieve the required sample size.

### COVID Appropriate Behaviors

COVID appropriate behaviors are preventive measures and practices to fight the spread of COVID 19. To be effective, in the long run, it is pertinent that every citizen follows it judiciously. The guidebook from the Ministry of Health and family welfare, Government of India, has a comprehensive list of fifteen preventive behavior practices to surmount the spread of the disease (MoHFW (GOI), 2020). In addition, we have added suggested measures that are under process by various organizations for the effective reopening of schools (IASC, 2020; UNESCO, 2020b; The Ministry of Home Affairs, 2020) .

### Operational Definitions and Study Groups

Public schools include schools that are managed/aided by the Government. The Central Board of Secondary Education (CBSE) is a national level board of education in India for public and private schools, managed by the Government of India. The Indian Certificate of Secondary Education (ICSE) is an examination conducted by the Council for the Indian School Certificate Examinations (private board of secondary education in India). The Karnataka state government manages the State Education Examination Board (State board). We grouped schools as public versus private, and school boards were grouped as CBSE+ ICSE versus state boards for comparison of mean perception scores of teachers.

### Data Collection

**Fig. 1:**
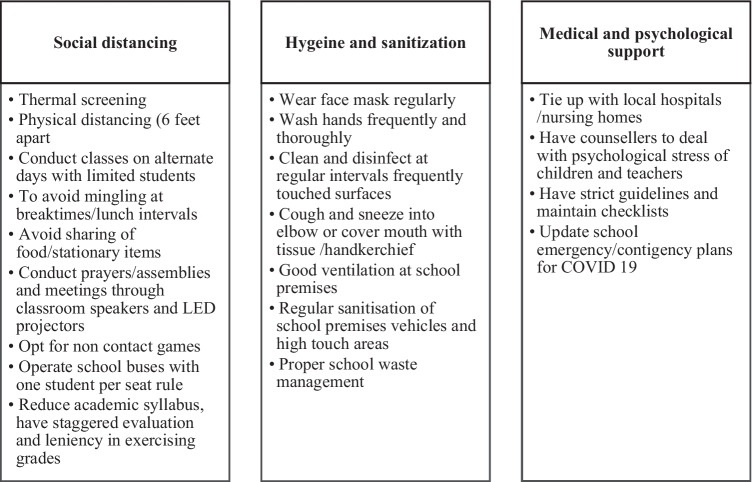
COVID APPROPRIATE BEHAVIOURS

We obtained permission from the Block Education Officer (BEO) and school authorities after explaining the details of the study. The link of the questionnaire by google forms was sent to school teachers via whatsApp or email. The questionnaire had four sections- section 1 included demographic data, section 2: social distancing measures, section 3: hygiene and sanitization measures, and section 4: medical and psychological support measures. School teachers who were willing to participate in the study were included. Teachers had to give their perceptions towards COVID appropriate behaviors (Fig. 1) on a symmetric agree-disagree scale (five-point Likert scale). As per the five-point Likert scale, one indicated strongly disagree, and five indicated strongly agree. The participant information sheet, which relates to the purpose of the study, was enclosed. Informed consent was collected from the teachers via google forms. Data was collected using a validated online questionnaire through google forms. The teacher’s opinions regarding the suggested measures under process by various organizations (IASC, 2020; UNESCO, 2020b; The Ministry of Home Affairs, 2020) were collected.

IBM SPSS Statistics analyzed data for Windows, Version 25.0, Armonk, NY: IBM Corp. Results were expressed as proportions using appropriate tables and figures. For comparison across the groups, independent sample’t’ test and chi-square tests were used. A ‘P’ value of <0.05 was considered statistically significant.

## Results

Out of the 547 schoolteachers, 481(87.9%) were females, 381 (69.6%) were between fourth and fifth decades, 469(85.7%) were from private institutions and 345(63.1%) from schools that had CBSE/ICSE boards.

### Perceptions on Social Distancing Measures

Ensuring different break times/intervals for children, avoiding mingling of children with no handshakes, high fives, and hugs were agreed by 97.5% of teachers. Following social distancing in labs with avoiding sharing of stationery items was consented by 95.8% of teachers. About 93% of teachers wanted a reduction in the academic syllabus. Distancing benches to maintain six feet distance between students, having staggered lunch breaks, preferably cooked food from home, and opting for non-contact games was opined by 91% of school teachers.

Around 90% of teachers agreed to adopt thermal screening at school, conduct school prayers /assemblies using classroom speakers, and have parent-teacher association (PTA) meetings by online mode. Among the teachers, 83.7% agreed to conduct alternate day classes with limited children. Nearly half (47.7%) concurred with teaching online courses and having glass partitions (cubicle type) for each student, while 30% disagreed for both. Three-fourths (75.7%) did not want to have reshuffling of children and teachers between classes. About 80.1% agreed to adopt online-offline integrated learning, while 86.3% wanted a staggered exam evaluation and leniency in awarding marks.

Around 96.3% felt there should be a supervised queue system for children to exit from school. About 94.5% felt that they should notify parents to avoid student overcrowding in respective private school buses. Operating school buses with one student per seat was concurred by 79% of teachers.

### Perceptions on Hygiene and Sanitization Measures

Regarding hygiene and sanitization, 94.5% and 93.3% of school teachers agreed that children should appropriately wear a facemask and follow proper handwashing techniques with soap and water. About 96.9% of teachers felt that children must cough/sneeze into elbow/ cover mouth, followed by handwashing. About 98.7% of teachers opined for having good ventilation in classrooms/school premises. Nearly 98.4% of school teachers admitted the need for adequate water supply in washrooms, 94.3% agreed to regularly sanitize school premises /school vehicles, especially high-touch areas with disinfectants, and 98%felt the need to ensure proper school waste management with safe disposal.

### Psychological and Medical Support Measures

About 98% of teachers admitted that they would set up strict rules not to attend schools if fever, cough, sore throat, or any illness were present in children. In addition, 94.5% felt the need to develop school emergency/contingency plans; 90.1% agreed to deal with psychological stresses related to the pandemic in children with counsellers and 93% opined to have  collaboration with local hospitals/nursing homes for managing medical emergencies.

### Mean Perception Scores of Teachers across Groups

The perceptions of school teachers towards the following variables, namely i) academic syllabus reduction, ii) integration of offline-online learning for children by using the cloud-based system and, iii) conducting parent-teacher association (PTA) meetings via online/other telecommunication channels, were found to be statistically significant (p value <0.05) when we made a comparison of the mean perception scores between private versus public schools (see Table 1& 2) as well as between the school boards (see Tables 3 & 4).

**Table 1:** Comparison of Perception Scores of Teachers towards Social Distancing Measures (Institution wise)

**Variable**	**School institution**		**P value**
	**Government and aided** **(n = 78)** **Mean(SD)**	**Private** **(n = 469)** **Mean(SD)**	
Thermal screening of children at entry and exit from school.	4.55 (0.92)	4.65 (0.83)	0.33
To conduct classes on alternate days /Double shift basis with limited number of students per class (15–20).	4.44 (1.04)	4.30 (1.05)	0.29
To conduct online classes for students.	3.21 (1.25)	3.47 (1.34)	0.11
Adopt a Cloud based system to integrate online offline learning for students.	3.96 (0.99)	4.32 (0.87)	0.001*
To arrange benches and desks so as to maintain six feet distance between students.	4.67 (0.57)	4.54(0.84)	0.10
To have glass partitions (cubicle type) for each seated student.	3.09 (1.45)	3.22 (1.42)	0.45
To reduce the academic syllabus for each class.	4.82 (0.50)	4.65 (0.67)	0.01*
Have different break times/intervals for each class, avoid mingling of students and ensure no handshakes, high fives and hugs.	4.59 (0.78)	4.52 (0.89)	0.53
As far as possible to avoid shuffling of students and their class teachers for the respective sections.	4.12 (1.08)	4.14 (1.12)	0.88
Have staggered lunch breaks with supervision; Have cooked food preferably from home; To avoid sharing of food.	4.72 (0.60)	4.60 (0.85)	0.14
Avoid sharing stationary items/books between students. And ensure social distancing during labs.	4.46 (0.94)	4.65 (0.76)	0.09
Conduct school prayers /assemblies/other common meetings for students through classroom speakers or LED projectors.	4.40 (1.09)	4.57 (0.94)	0.14
Opt for non-contact games and activities like yoga, hopscotch, aerobics, etc., or minimal contact games like badminton, table tennis; all under supervision.	4.62 (0.69)	4.50 (0.89)	0.21
Operate school buses with one student per seat rule.	4.19 (1.14)	4.29 (1.02)	0.45
Notify parents to avoid overcrowding in private school vehicles arranged by them	4.79 (0.54)	4.70 (0.68)	0.15
To have a supervised queue system for children to exit out from school with no crowding of parents within the school premises.	4.74 (0.57)	4.78 (0.56)	0.57
Parent teacher association (PTA) meetings to be conducted online or through other telecommunication channels.	4.27 (0.96)	4.62 (0.71)	0.002*
Have staggered evaluation and should be lenient in exercising grades for student’s attendance and test marks this year	4.35 (0.79)	4.40 (0.81)	0.81

*P values by independent sample‘t’ test;< 0.05 is significant,

**Table 2:** Comparison of Perception Scores of Teachers towards Hygiene, Sanitization, Psychological and Medical Support Measures (Institution wise)

**Variable**	**School institution**		P value*
	**Government and aided** **(n = 78)** **Mean(SD)**	**Private** **(n- 469)** **Mean(SD)**	
Special attention at the entrance of school and classrooms to promote hand hygiene.	4.77 (0.58)	4.78 (0.54)	0.89
Children should wear face mask in the appropriate manner.	4.71 (0.82)	4.78 (0.66)	0.36
Children should do proper handwashing with soap and water (for at least minimum of 20 s).	4.76 (0.69)	4.70 (0.70)	0.47
Children should cough and sneeze into the elbow or cover mouth with a tissue/handkerchief.	4.85 (0.56)	4.82 (0.59)	0.68
Children need to wash hand after coughing, sneezing, rubbing of nose, etc..	4.74 (0.65)	4.77 (0.63)	0.74
Good ventilation to be present in the classrooms and school premises.	4.83 (0.57)	4.88 (0.40)	0.39
Ensure adequate water supply in washrooms for students.	4.87 (0.54)	4.90 (0.41)	0.60
Promote and demonstrate the correct method of handwashing with soap and water and use of masks-by conducting demonstrative classes by teachers.	4.78 (0.60)	4.76 (0.61)	0.78
Regular sanitization of the school vehicles and school premises especially high touch areas like handles, seats, windows, doorknobs, desks and benches with disinfectants (at least once a day).	4.63 (0.69)	4.74 (0.61)	0.17
Ensure proper school waste management with safe disposal on daily basis.	4.87 (0.44)	4.84 (0.47)	0.57
Have hand washing stations with soaps water/alcohol based hand rub/sanitizer in each classroom and washrooms entrances and exits.	4.62 (0.79)	4.62 (0.75)	0.92
To ensure necessary stock of disinfectants, hand rubs, sanitizers etc. to be present in the school.	4.81 (0.54)	4.76 (0.62)	0.51
Will make strict rules not to send children to school if symptoms of fever, cough, sore throat or any illness.	4.95 (0.22)	4.90 (0.40)	0.13
Once well, to rejoin back to class with medical certificate.	4.50 (0.82)	4.77 (0.64)	**0.01** *
Have counselors to deal with psychological stress the child is going through the COVID 19 time.	4.40 (0.84)	4.54 (0.78)	0.78
The school should update or develop school emergency and contingency plans during this COVID 19 time.	4.55 (0.66)	4.68 (0.50)	0.50
Have a tie-up with local hospital/nursing homes; on campus/on call availability of medical attendants.	4.60 (0.61)	4.62 (0.71)	0.71
To maintain the checklist of guidelines being followed on daily basis.	4.54 (0.68)	4.58 (0.69)	0.69

*P values by independent sample‘t’ test;< 0.05 is significant

**Table 3:** Comparison of Perception Scores of Teachers towards Social Distancing Measures (School board wise)

**Variable**	**School board**		P value*
	**CBSE + ICSE** **(n-346)** **Mean(SD)**	**State board** **(n- 201)** **Mean(SD)**	
Thermal screening of children at entry and exit from school.	4.66 (0.81)	4.60 (0.91)	0.45
To conduct classes on alternate days /Double shift basis with limited number of students per class (15–20).	4.23 (1.1)	4.48 (0.91)	0.005*
To conduct online classes for students.	3.50 (1.33)	3.31 (1.32)	0.10
Adopt a Cloud based system to integrate online offline learning for students.	4.33 (0.86)	4.17 (0.93)	0.04*
To arrange benches and desks so as to maintain six feet distance between students.	4.51 (0.86)	4.65 (0.69)	0.041*
To have glass partitions (cubicle type) for each seated student.	3.21 (1.41)	3.19 (1.46)	0.86
To reduce the academic syllabus for each class.	4.63 (0.67)	4.76 (0.60)	0.025*
Have different break times/intervals to be made for each class, avoid mingling of students and ensure no handshakes, high fives and hugs.	4.51 (0.91)	4.56 (0.82)	0.54
As far as possible to avoid shuffling of students and their class teachers for the respective sections.	4.14 (1.10)	4.13 (1.18)	0.95
Have staggered lunch breaks with supervision; Have cooked food preferably from home; To avoid sharing of food.	4.65 (0.72)	4.57 (0.95)	0.30
Avoid sharing stationary items/books between students.	4.67(0.72)	4.54 (0.91)	0.09
Conduct school prayers /assemblies/other common meetings for students though classroom speakers or LED projectors.	4.58 (0.92)	4.49 (1.03)	0.32
Opt for non-contact games and activities like yoga, hopscotch, aerobics, etc., or minimal contact games like badminton, table tennis; all under supervision.	4.52 (0.92)	4.52 (0.77)	0.95
Operate school buses with one student per seat rule.	4.27 (1.03)	4.27 (1.07)	0.99
Notify parents to avoid overcrowding in private school vehicles arranged by them.	4.68 (0.66)	4.76 (0.67)	0.21
To have a supervised queue system for children to exit out from school with no crowding of parents within the school premises.	4.78 (0.57)	4.77 (0.56)	0.73
Parent teacher association (PTA) meetings to be conducted online or through other telecommunication channels.	4.64 (0.71)	4.46 (0.83)	0.01*
Have staggered evaluation and should be lenient in exercising grades for student’s attendance and test marks this year.	4.40 (0.80)	4.36 (0.81)	0.56

*P values by independent sample‘t’ test;< 0.05 is significant,

CBSE: Central Board of Secondary Education; ICSE: Indian Certificate of Secondary Education

LED: Light Emitting Diode

**Table 4:** Comparison of Perception Scores of Teachers towards Hygiene, Sanitization, Psychological and Medical Support Measures (School board wise)

**Variable**	**School board**		P value*
	**CBSE + ICSE** **(n-346)** **Mean(SD)**	**State board** **(n- 201)** **Mean(SD)**	
Special attention at the entrance of school and classrooms to promote hand hygiene.	4.78 ± 0.52	4.77 ± 0.58	0.72
Children should wear face mask in the appropriate manner.	4.78 ± 0.64	4.75 ± 0.76	0.60
Children should do proper handwashing with soap and water (for at least minimum of 20 s).	4.69 ± 0.71	4.72 ± 0.68	0.65
Children should cough and sneeze into the elbow or cover mouth with a tissue/handkerchief.	4.79 ± 0.62	4.87 ± 0.51	0.17
Children need to wash hand after coughing, sneezing, rubbing of nose, etc..	4.75 ± 0.68	4.79 ± 0.55	0.48
Good ventilation to be present in the classrooms and school premises.	4.87 ± 0.43	4.88 ± 0.42	0.88
Ensure adequate water supply in washrooms for students.	4.89 ± 0.44	4.90 ± 0.42	0.85
Promote and demonstrate the correct method of handwashing with soap and water and use of masks-by conducting demonstrative classes by teachers.	4.77 ± 0.63	4.76 ± 0.58	0.82
Regular sanitization of the school vehicles and school premises especially high touch areas like handles seats windows, doorknobs, desks and benches with disinfectants (at least once a day).	4.76 ± 0.58	4.66 ± 0.69	0.08
Ensure proper school waste management with safe disposal on daily basis.	4.82 ± 0.49	4.88 ± 0.40	0.14
Have hand washing stations with soaps water/alcohol based hand rub/sanitizer in each classroom and washrooms entrances and exits.	4.64 ± 0.73	4.60 ± 0.80	0.53
To ensure necessary stock of disinfectants, hand rubs sanitizers etc. to be present in the school.	4.76 ± 0.62	4.78 ± 0.58	0.66
Will make strict rules not to send children to school if symptoms of fever, cough, sore throat or any illness.	4.89 ± 0.43	4.95 ± 0.25	0.08
Once well, to rejoin back to class with medical certificate.	4.75 ± 0.69	4.71 ± 0.64	0.53
Have counselors to deal with psychological stress the child is going through the COVID 19 time.	4.58 ± 0.76	4.43 ± 0.85	0.04*
The school should update or develop school emergency and contingency plans during this COVID 19 time.	4.68 ± 0.58	4.62 ± 0.65	0.24
Have a tie-up with local hospital/nursing homes; on campus/on call availability of medical attendants.	4.67 ± 0.62	4.52 ± 0.81	0.019*
To maintain the checklist of guidelines being followed on daily basis.	4.58 ± 0.69	4.55 ± 0.69	0.55

*P values by independent sample‘t’ test;< 0.05 is significant,

CBSE: Central Board of Secondary Education; ICSE: Indian Certificate of Secondary Education

LED: Light Emitting Diode

In addition, measures such as i)conducting classes on alternate days /double shift basis with a limited number of children per class (15 to 20), ii) arranging benches and desks to maintain six feet distance between children, iii) dealing with psychological stress with counselors, iv) having a tie-up with local hospital/nursing homes and, v) arranging on-campus/on-call availability of medical attendants were found to be statistically significant (p value <0.05) when the mean perceptions scores across school boards were compared (see Tables 3 & 4).

## Discussion

In our study, teachers perceived that adopting social distancing and staggered children’s joining in phases with appropriate safe, hygienic handwashing practices would be safer and more beneficial. The majority of school teachers strongly agreed to implement safe practices.

COVID appropriate behaviors like proper handwashing with soap and water, avoiding sharing food items, wearing a facemask, washing hands regularly were consented to by most teachers. Likewise, ensuring good ventilation in classrooms, covering their mouth while coughing/sneezing followed by handwashing, was agreed by the teachers in our study.

A study among Taiwanese teachers noted that higher risk perception among them had a higher implementation of preventive measures such as frequent handwashing, measuring body temperature, conducting classes with open windows, and wearing masks and was similar to our study findings. However, using hand sanitizer and disinfection of workplaces daily was lower than levels perceived by our teachers. (Tang et al., 2021).

Earlier studies among university students (Vande Velde et al., 2021; Mant et al., 2021) and respondents at different workplaces in China (Tan et al., 2020) showed willingness to adopt preventive behaviors. Among adults in Australia, 84.9% of respondents reported undertaking≥1 of the three hygiene-related behaviors, the most common being washing hands with soap and water. About 93.4% agreed to undertake ≥one of the six avoidance-related behaviors, the most common being avoiding crowded places (Seale et al., 2020).

The COVID 19 snapshot monitoring (COSMO) study among the German men and women documented that COVID protective behavior was influenced by the COVID 19 related knowledge and risk perception. (Rattay et al., 2021). Our study did not assess the teacher’s knowledge and risk perceptions related to COVID 19.

In this study, the difference in the perception scores between private and public schools could be because many respondents were from private schools and possibly because of practical issues related to the lack of infrastructure faced by the public school teachers. However, we could not assess this aspect personally in the current pandemic situation.

Earlier studies on influenza epidemics have demonstrated the efficacy of social distancing measures and school closures (Jackson et al., 2014). School closures aid in breaking the social contacts between the households and suppress community transmission (Cowling et al., 2008). With the social distancing measures adopted for COVID 19 outbreaks, there was a reduction in community transmission by 44%, which was much more than the estimated 10–15% reduction in transmission of influenza illness by the implementation of school closure alone during the 2009 pandemic in Hong Kong (Cowling et al., 2008).

During COVID 19 outbreaks in the United Kingdom, isolated school closure was predicted to decrease total deaths by two to 4 %. More effective would be case isolation as a lone measure, while the most effective results would be if combined with population-wide social distancing, school and university closures. The modeling study concluded that isolated school closures are insufficient to mitigate the COVID 19 pandemic, this being in contrast to seasonal influenza epidemics (Ferguson et al., 2020).

Less drastic than school closure is potential social distance measures such as shortening the school week, distancing the students in classes, staggering the school start, lunch, and break times across classes, canceling nonessential activities and meetings. However, there is limited literature on such school practices that advocate social distancing as per the reviews on influenza pandemics (Uscher-Pines et al., 2018). In Taiwan, during the H1N1 influenza pandemic, suspensions of classes instead of school closures were done. The students sat in a homeroom class with a core teacher while other teachers moved between the classes; this reduced the social disruption between children and acted as an excellent social distance measure (Yen et al., 2014). A systematic review concluded that policymakers should consider combinations of social distance measures at schools rather than the school’s closure. Less disruptive social distancing interventions in the school need to be considered for extended periods (Viner et al., 2020).

There was an increase in outpatient visits for influenza-like illness (ILI) after an average of fourteen days of school reopening because of transmission surges of influenza (Chao et al., 2010; Cowling et al., 2008). In a modeling study conducted in Wuhan, China, there was a reduction in the final size and peak incidence of the COVID 19 outbreak by adopting a package of social distancing measures. There was a second peak of infection with earlier relaxation (after two months’ restriction) of social distancing measures, and with three months’ restrictions, there was no peak noted. It stressed that gradual relaxation of social distance measures and staggered joining of people to workplaces would make a difference in the pandemic. The same could be applied towards school reopening (Prem et al., 2020).

After the first wave of COVID 19, several countries with their government officials have reopened schools worldwide with safety guidelines to have social distancing, good hygiene, and sanitization. In addition, organizations such as WHO, UNICEF, UNESCO, etc., were helping to streamline school reopening (IASC, 2020; UNESCO, 2020b; The Ministry of Home Affairs, 2020). Denmark was the first to reopen schools from April 15th, 2020, using the pod method, where the young children who arrived were divided into micro-groups or pods (Express Web Desk, , 2020). Norway and a few other counties adopted the cohort method (Godin, 2020). In either of the strategies adopted, children had different arrival/lunchtime with no interactions, different play, and work zones, maintenance of social distancing, school desks and benches well distanced, classrooms well ventilated, regular sanitization of classrooms and washrooms, regular handwashing, and usage of face masks (Express Web Desk,, 2020; Godin,Godin, 2020).

In China, the ministry of education had temperatures of children checked on arrival to school. They attended schools if China’s smartphone health code program displayed a green code of health. Students of Yang Zheng Primary school in Hangzhou adopted wearing a do-it-yourself (DIY) one-meter hat while attending school to acclimatize themselves towards social distancing (“Coronavirus-Schools start,” , 2020).

India experienced the first wave of COVID 19 Infection since March 2020. Indian schools have had partial reopening since the fourth week of September 2020 for classes 9th to 12th on a choice basis for doubt clarification and guidance (Ojha, 2020). As per guidelines, Karnataka had school reopening for higher courses since January 2021 with no significant surge of cases (Ghosh, 2020).

The reopening of schools in 22 European countries did not cause a significant rise in coronavirus infections among children (Chopra, 2020). Similarly, a study that analyzed data from 191 countries documented no correlation between surges in COVID 19 cases after reopening schools (Basu, 2020). On the contrary, in a recent study that investigated the transmissibility of COVID 19 at six elementary schools of the Georgia district, nine clusters of COVID 19 cases were found in 32 students and 13 educators. Inadequate mask usage and not maintaining adequate social distancing measures were noted in these clusters (Gold et al., 2021). India currently had the second wave of COVID 19 infection, with surges of cases increasing from mid-March 2021 onwards. Thus it was decided to keep schools closed (Ghosh, 2021), along with lockdown in various states. Currently, the schools have reopened for multiple classes in a phased manner in India.

As per the revised guidelines on the National COVID vaccination program (MOHFW (GoI), 2021), vaccine administration was prioritized to high-risk populations and senior citizens age groups. Depending on the vaccine supply at the centers, the vaccines were administered. The teachers are currently undergoing and completing their second doses of vaccine, and hence the data on vaccination status was not included in our study. Teachers’ vaccination will help in the renormalization of in-person teaching /learning methods at school. The COVID 19 vaccination for children between 12 to 18 years of age is planned to be implemented from October 2021.

As a necessity measure during the pandemic, there was a surge in telehealth usage for easy and safe access to health care delivery. Telehealth services in the school premises will bridge the gap in healthcare accessibility and elude the repercussions of poor health on the child’s education and academic outcomes. For schoolchildren, especially in rural areas, timely access to healthcare is a critical priority (India Today Web Desk, 2021). A recent review has shown telehealth usage in pediatric occupational therapy is an alternative service delivery model that facilitates access for children and families to rehabilitation services (Önal et al., 2021).

School-based telehealth services focus on immediate primary health care and manage chronic diseases, speech therapy, counseling on nutritional, mental, and behavioral health issues, dental screenings, and health education. School-based telehealth centers have the prospects to reach out to larger pediatric populations who require health care because of COVID-19-related lapses in services and to address COVID-19-related health issues as schools reopen. (Williams et al., 2021).

Integrated medical services by a team of counselors, pediatricians, nurses, and medical practitioners through school-based health centers (SBHCs) are rampant in the United States (India Today Web Desk,2021). In India, a pilot project of setting up school health clinics in fifteen government schools in Delhi is under process to provide telehealth services that will address the issues related to schoolchildren’s physical and mental health, especially during times when the schools are planning to reopen. (TNN, 2021).

The study’s limitations were i) observational study with a higher representation of private schools, ii) vaccination status of teachers was not available at time of the study, iii) chances of response bias since it is a questionnaire-based via google docs form and findings cannot be generalized. Further effectiveness of these preventive measures being implemented appropriately needs to be studied across various countries.

## Conclusion

This research finds the teachers to accept the suggested COVID appropriate behaviors as protective mitigative measures to manage children at school once it reopens, implying its feasibility to have smooth functioning of the school and prevent the infection surges. In addition, they are keen on having medical and psychological supportive measures that will aid the teachers, students, and families.

The teacher’s perceptions on COVID appropriate behavioral measures are essential for policy perspectives and academics in the long run. The opinions from teachers will inform the policymakers to emphasize setting up practical mitigating guidelines in schools and educational institutes to control the spread of the disease. Teachers’ perceptions of preventive measures can be used as a reference/strength to tackle epidemics due to any emerging infectious diseases in the future.
